# Image-Evoked Emotion Recognition for Hearing-Impaired Subjects with EEG Signals

**DOI:** 10.3390/s23125461

**Published:** 2023-06-09

**Authors:** Mu Zhu, Haonan Jin, Zhongli Bai, Zhiwei Li, Yu Song

**Affiliations:** Tianjin Key Laboratory for Control Theory and Applications in Complicated Systems, School of Electrical Engineering and Automation, Tianjin University of Technology, Tianjin 300384, China; zm78792021@163.com (M.Z.); tianjinjhn1231@hotmail.com (H.J.); zl.bai@hotmail.com (Z.B.)

**Keywords:** EEG signals, emotion faces, emotion classification, hearing-impaired subjects, self-attention mechanism

## Abstract

In recent years, there has been a growing interest in the study of emotion recognition through electroencephalogram (EEG) signals. One particular group of interest are individuals with hearing impairments, who may have a bias towards certain types of information when communicating with those in their environment. To address this, our study collected EEG signals from both hearing-impaired and non-hearing-impaired subjects while they viewed pictures of emotional faces for emotion recognition. Four kinds of feature matrices, symmetry difference, and symmetry quotient based on original signal and differential entropy (DE) were constructed, respectively, to extract the spatial domain information. The multi-axis self-attention classification model was proposed, which consists of local attention and global attention, combining the attention model with convolution through a novel architectural element for feature classification. Three-classification (positive, neutral, negative) and five-classification (happy, neutral, sad, angry, fearful) tasks of emotion recognition were carried out. The experimental results show that the proposed method is superior to the original feature method, and the multi-feature fusion achieved a good effect in both hearing-impaired and non-hearing-impaired subjects. The average classification accuracy for hearing-impaired subjects and non-hearing-impaired subjects was 70.2% (three-classification) and 50.15% (five-classification), and 72.05% (three-classification) and 51.53% (five-classification), respectively. In addition, by exploring the brain topography of different emotions, we found that the discriminative brain regions of the hearing-impaired subjects were also distributed in the parietal lobe, unlike those of the non-hearing-impaired subjects.

## 1. Introduction

Emotion is the attitude and experience generated after human beings compare objective things with their needs. It reflects people’s current physiological and psychological state, which plays a crucial role in people’s cognition, communication, and decision making [1]. Researchers believe that any emotion produced by human beings must be accompanied by some physical changes, such as facial expression, muscle contraction and relaxation, and visceral activities. How to understand and express emotions is affected by a human’s ability to perceive the outside world and express themselves.

With the development of emotional computing and artificial intelligence, the study of emotion recognition via electroencephalogram (EEG) has been paid more attention. In recent years, EEG-based emotion recognition has mainly focused on healthy people and achieved good results [2]. However, few studies have examined groups such as hearing-impaired people [3,4]. Some researchers believe that due to hearing loss, hearing-impaired people find it difficult to receive information from the outside world as fully and accurately as hearing people [5,6]. As a result, they may have cognitive bias which can cause them to interpret interactions excessively negatively, producing interpersonal cognitive bias [7]. Therefore, it is of great importance to study the facial emotion recognition ability of hearing-impaired people to examine and, if desired by the individuals concerned, aid their social adaptability. In order to aid the facial recognition ability of hearing-impaired subjects where desired, it is necessary to understand the differences in their recognition of the emotions of different faces. Therefore, in this paper, the emotions of non-hearing-impaired subjects and hearing-impaired subjects were induced by Chinese pictures of faces showing certain emotions, and their EEG signals were collected for emotion recognition to analyze the cooperative working mechanisms of the brain regions of hearing-impaired subjects when recognizing facial emotions.

Feature extraction is particularly important in EEG emotion recognition [7]. EEG signals comprise high-dimensional temporal data with a low signal–noise ratio [8,9]. Therefore, after effective feature extraction, EEG features containing rich information can improve the ability to distinguish types of emotion in limited-feature spatial dimensions [10]. Time domain features were first used because of their strong intuitiveness and good recognition effect for specific waveform signals. Fourati et al. [11] proposed the echo state network (ESN), which used recurrent layers to project original EEG signals into high-dimensional state space, and achieved satisfactory results. Frequency domain features are based on Fourier transform, and the distribution of signal power along frequency is obtained by transforming the time domain to the frequency domain. This has been proven to be efficient in EEG emotion recognition using methods such as power spectral density (PSD) and differential entropy (DE) [12]. Duan et al. [13] applied DE features to EEG signals in emotion recognition and found that DE features had a high recognition rate and stability for positive and negative emotion recognition. Li et al. [14] proposed a spatial feature extraction method to capture spatial information between different channels using a hierarchical convolutional neural network based on a two-dimensional EEG graph. Although existing emotion recognition methods have achieved high accuracy, most of them only consider a single feature or a combination of two features, ignoring the complementarity between different features.

EEG emotion recognition is essentially a pattern recognition problem aiming to embed highly discriminative emotion features into EEG signals and improve the accuracy of emotion recognition. Early emotion recognition models are mainly shallow machine learning models such as support vector machine [15] (SVM), the k-nearest neighbor algorithms [16] (KNN), and so on. Among them, the SVM algorithm is widely used because of its unique advantages in solving small-sample, high-dimension, and nonlinear machine learning tasks. Bhardwaj et al. [17] extracted PSD features and used SVM and the linear discriminative analysis (LDA) algorithm for emotion recognition. The results show that SVM is superior to LDA in emotion classification. In terms of deep learning, Cimtay et al. [18] proposed using a pre-trained CNN to extract features, which eliminates the influence of voltage amplitude fluctuation through data normalization and adds a pooling layer and a fully connected layer to the pre-trained network, thus improving the classification performance of the network. Song et al. [19] further modeled multichannel EEG data into graph data with the basic units of an electroencephalogram, providing a new perspective for analyzing EEG data. They designed the Dynamic Graph Convolutional Neural Network (DGCNN). Based on the learned adjacency matrix, the model can complete the feature propagation between electrodes and learn more discriminative features to improve emotion recognition. Previous studies have only focused on local features affecting the capacity and generalization of the whole model but ignored global features. Therefore, starting from effectively integrating global and local interactions, this paper focuses on balancing model capacity and universality in order to improve model performance.

In this paper, we selected the Chinese-style facial expression pictures from CFAPS as stimuli to induce subjects’ emotions by displaying corresponding facial expression pictures. Finally, 20 non-hearing-impaired subjects and 20 hearing-impaired subjects were collected based on EEG induced by five emotional images. Besides, the multi-axis self-attention module composed of the window attention module and grid attention module effectively combines local and global features to improve model classification performance and reduce model computational complexity. Promising classification results are obtained on the datasets of hearing-impaired subjects and non-hearing-impaired subjects, respectively, which proves the validity of the classification model. By drawing a brain topographic map based on DE features, we further compare and analyze the differences in brain region energy in emotion recognition between hearing-impaired subjects and non-hearing-impaired subjects. Compared with non-hearing-impaired subjects, the changes in the emotion recognition of hearing-impaired subjects are not only concentrated in the temporal lobe but also distributed in the parietal lobe. The main contributions of this study are given as follows.

(1) We have constructed an emotional EEG dataset using facial expression picture stimuli, featuring both non-hearing-impaired and hearing-impaired subjects.
(2) To facilitate fusion, we have developed two novel constructs—the subtract symmetric matrix (SSM) and the quotient symmetric matrix (QSM)—based on the original signal and DE feature. These matrices have been designed keeping in mind the electrode positions in the widely recognized international 10–20 system, and take into account the nature of the electrode pairs in symmetrical positions. SSM quantifies the difference between the eigenvalues of the left and right brain regions by measuring the characteristic differences of 27 pairs of symmetric electrodes. Similarly, QSM computes the difference between the eigenvalues of the left and right regions of the brain using the same methodology.
(3) Our work puts forth a groundbreaking multi-axis self-attention mechanism for the recognition of emotions through EEG among both non-hearing-impaired and hearing-impaired individuals. Our multi-axis self-attention mechanism is composed of two modules that run in parallel: a window attention module which focuses on local features of the EEG signal, and a global attention module which extracts the global features.

The rest of the paper is summarized as follows. In Section 2, we introduce the experiment setup in detail. Section 3 describes the proposed feature extraction and emotion classification method. The experimental results are analyzed in Section 4. Section 5 discusses this work. Finally, we summarize the paper and look forward to future research development in Section 6.

## 2. Materials

Current methods of emotion induction mainly stimulate subjects through pictures [20], audio [21], and video [22] to obtain corresponding emotional EEG signals. According to the mirror neuron theory [23], when people observe another person performing a certain activity, their own brain activity is also as if they were performing the process. In addition, the mirror neuron system is also involved in recognizing emotions through other people’s facial expressions and gestures. Therefore, this experiment is designed to induce corresponding emotions by showing facial expressions of different emotions to subjects. The EEG signal of 20 hearing-impaired college students and 20 non-hearing-impaired college students were collected when they viewed the pictures of five different emotional faces under the same experimental materials and environment. Experimental preparation mainly includes recruiting subjects and selecting stimulus materials, and introducing experimental procedures. The Ethics Committee of Tianjin University of Technology has approved this experiment for emotion recognition research (May 2022).

### 2.1. Subjects

In this work, twenty subjects (7 females and 13 males) with an average age of 22 years old were recruited from the School of Audiology and Artificial Sciences of Tianjin University of Technology for an emotion induction experiment. We only conducted subject-dependent experiments—the training dataset and test dataset were from the same subject—so that gender did not influence the experimental results. Basic personal information and auditory information were collected before participating in the experiment. The subjects had uncorrected or corrected vision, were right-handed, and had no history of mental illness. All of the subjects had hearing loss in both ears, 4 subjects had congenital disorders, and 18 subjects wore hearing aid devices. The situation of 20 hearing-impaired subjects was shown in Table 1. In addition, we also recruited 20 students (14 males and 6 females, aged 18–25 with an average age of 22) from the School of Electrical Engineering and Automation to participate in the experiment. The 20 subjects with unimpaired hearing were right-handed and had no history of mental illness. The 40 participants all provided informed consent approved by the Ethics Committee of Tianjin University of Technology. Before the experiment, all subjects were informed about the purpose of the experiment and the harmlessness of the EEG collection equipment. If there were special circumstances, including but not limited to fatigue, environmental suitability, and discomfort caused by the stimulus images, all subjects were permitted to terminate and withdraw from the experiment.

### 2.2. Stimulation Materials Selection

In our previous research, we have found that small changes in the facial expression of hearing-impaired subjects during video stimuli watching are particularly indicative of emotional changes. In order to verify whether this conclusion is still valid under different emotional induction methods, this study used facial expression pictures as stimuli, which contain rich information about facial changes.

**Figure 1:**
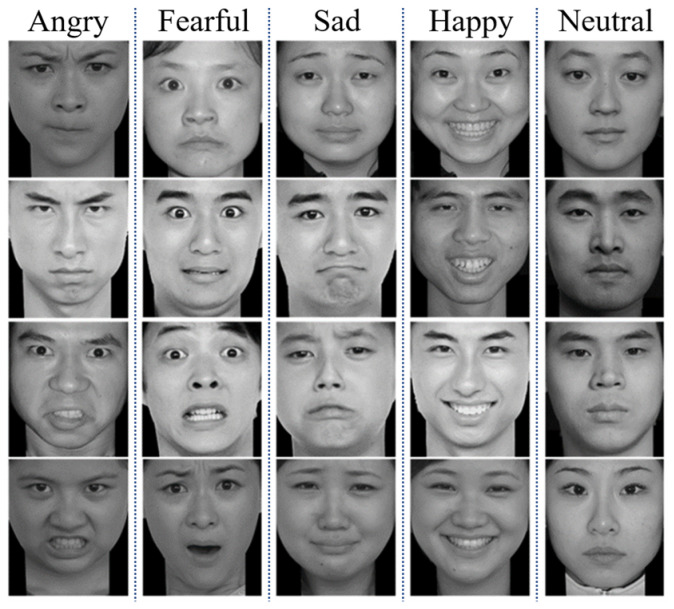
The schematic diagram of the experimental paradigm flow.

The stimulus material of the experiment was 240 emotional images of faces selected from the Chinese Facial Affective Picture System [24] (CFAPS). CFAPS, as a localized facial expression image system, contains a total of 870 facial expression images of seven emotional types. A total of 100 college students evaluated the emotional type of each image and give a score of 1–9 on the emotional intensity expressed based on the assessed emotional type. To ensure that the emotional images of faces in the system had a better effect on emotional arousal and practicability, we selected 5 types of facial expression pictures, including 36 angry faces, 36 fearful faces, 36 sad faces, 36 neutral faces, and 36 happy faces, as experimental stimulus materials. The emotional diagram of faces is shown in Figure 1. All images had a resolution of 260 × 300, and we used a 17-inch monitor to display emotional images.

With reference to the SEED dataset [25] our dataset can be tested for three-classification. We assign sadness to negative emotions, happiness to positive emotions, and neutral to neutral emotions. The using of more images of sad, neutral, and happy expressions is to increase the sample size in the three-classification task. No clothing or accessories were included in the images, and the parameters remained the same for all faces in the experiment.

### 2.3. Experimental Paradigm

In this experiment, the NeuSen W 364 EEG acquisition system developed by Neuracle (Changzhou, China), was used to collect the subjects’ EEG signals. The device collects EEG signals at a sampling rate of 1 k Hz and has 64 electrode channels. According to the international 10–20 system, TP9 and TP10 are used as reference electrodes for bilateral mastoid sensors.

**Figure 2:**
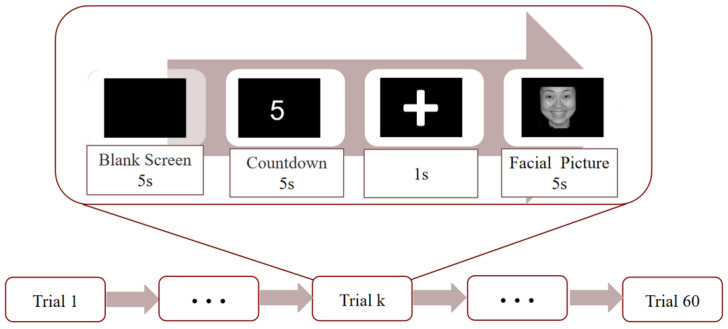
Emotional experiment paradigm.

The experiment was conducted in an isolated environment. Figure 2 shows the experimental paradigm. First, subjects were shown a black screen for 5 s to allow them to calm down their emotions and adjust their state. A five-second countdown was shown to prepare the subjects, and a cross was shown to remind them to pay attention. An emotional picture of a face was then displayed for five seconds and the subjects’ EEG signals were recorded. A test was conducted before the formal experiment, all subjects can ask questions at any time. The experimenters were prepared to help solve any problems that may arise during the experiment. The test procedure was the same as the formal experiment. Subjects were advised to stay as still as possible, understand emotions rather than simply mimic facial expressions, and try not to blink when the picture was shown, which would help to obtain noiseless EEG signals. The experiment was divided into four rounds; 60 trials were conducted in each round, among which 15 trials were in one group. After finishing each group of experiments, there would be 1–2 min of rest time and 5 min of rest time between each round of experiments.

## 3. Methods

The collected EEG signal needed to be preprocessed, and the input of the deep learning model can be obtained by feature extraction. Four eigenmatrices (SSM(OEF), QSM(OEF), SSM(DE), QSM(DE)) were extracted based on the asymmetric characteristics of the left and right brain regions. The model proposed in this paper first fuses four feature matrices and then obtains two deep features through local and global attention modules, respectively. The two deep features are fused again to obtain the feature vector with global and local representativeness, which is sent into the full connection layer to get the classification output of the model.

This section introduces the EEG signal pre-processing method, EEG emotion feature extraction method, and the construction of a classifier.

### 3.1. Data Pre-Processing

The EEG signals collected by experiments are inevitably mixed with various interference signals and artifacts, which need to be removed by signal pre-processing to improve the performance of subsequent emotion recognition. We used the EEGLAB toolbox to process the EEG signal. Firstly, the original EEG signal was down-sampled to 200 Hz and then we removed low-frequency drift and high-frequency noise by 1–75 Hz band-pass filter, and power frequency interference was eliminated by 49–51 HZ band-pass filtering. The bilateral mastoids of TP9 and TP10 were used as references, and then we interpolated the data to repair bad derivatives. Finally, independent component analysis (ICA) was used to remove artifacts.

### 3.2. Feature Extraction

**Figure 3:**
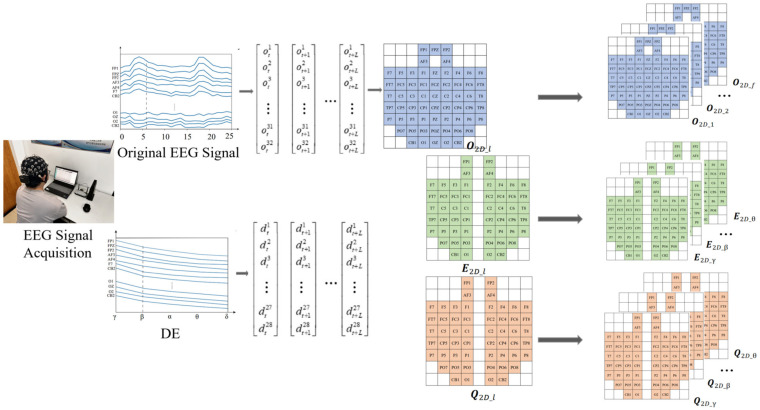
The construction process of feature matrix.

It is necessary to extract features from the pre-processed EEG signals to characterize the emotion-related information in the EEG signals. In this section, four different feature matrices (subtract symmetry and quotient symmetry matrix of the original signal and DE feature) will be constructed according to electrode positions in the international 10–20 system as the input of the classification model. The construction process of the eigenmatrix is shown in Figure 3.

In this paper, $O^{T}=\left(O_{1}^{T},O_{2}^{T},\ldots ,O_{T}^{T}\right)\in R^{E\times T}$ is defined as the EEG sample containing time *T*, *E* (=62) is the number of electrodes, and $O_{t}^{T}=\left(o_{t}^{1},o_{t}^{2},\ldots ,o_{t}^{E}\right)\in R^{E}\left(t\in \left\{1,2,\ldots ,T\right\}\right)$ represents the EEG signal of all electrodes collected at time *T*. Then, $O_{t}^{T}$ will be converted into a two-dimensional time-domain matrix $M_{t}^{T}\in R^{H\times W}$, namely, the 9 × 9 matrix we construct, as shown in the following formula:

(1) $$M_{t}^{T}=\left[\begin{array}{ccccccccc} 0 & 0 & 0 & o_{t}^{1} & o_{t}^{2} & o_{t}^{3} & 0 & 0 & 0\\ 0 & 0 & 0 & o_{lt}^{4} & 0 & o_{lt}^{5} & 0 & 0 & 0\\ o_{t}^{6} & o_{t}^{7} & o_{t}^{8} & o_{t}^{9} & o_{t}^{10} & o_{t}^{11} & o_{t}^{12} & o_{t}^{13} & o_{t}^{14}\\ o_{t}^{15} & o_{t}^{16} & o_{t}^{17} & o_{t}^{18} & o_{t}^{19} & o_{t}^{20} & o_{t}^{21} & o_{t}^{22} & o_{t}^{23}\\ o_{t}^{24} & o_{t}^{25} & o_{t}^{26} & o_{t}^{27} & o_{t}^{28} & o_{t}^{29} & o_{t}^{30} & o_{t}^{31} & o_{t}^{32}\\ o_{t}^{33} & o_{t}^{34} & o_{t}^{35} & o_{t}^{36} & o_{t}^{37} & o_{t}^{38} & o_{t}^{39} & o_{t}^{40} & o_{t}^{41}\\ o_{t}^{42} & o_{t}^{43} & o_{t}^{44} & o_{t}^{45} & o_{t}^{46} & o_{t}^{47} & o_{t}^{48} & o_{t}^{49} & o_{t}^{50}\\ 0 & o_{t}^{51} & o_{t}^{52} & o_{t}^{53} & o_{t}^{54} & o_{t}^{55} & o_{t}^{56} & o_{t}^{57} & 0\\ o_{t}^{58} & 0 & 0 & o_{t}^{59} & o_{t}^{60} & o_{t}^{61} & 0 & 0 & o_{t}^{62} \end{array}\right]$$

Differential entropy (DE) is a feature extraction method widely used in the field of EEG emotion recognition. It is an extension of Shannon entropy. For an EEG signal with a probability density function, DE can be approximately equal to the logarithm of the power spectrum at a certain frequency band. The calculation formula of electroencephalogram DE is as follows:

(2) $$h_{i}\left(X\right)=-\int_{X}\;f\left(x\right)\log\left(f\left(x\right)\right)dx=\frac{1}{2}\log\left(2\pi e\sigma_{i}^{2}\right)$$

where $f\left(x\right)$ is the probability density function obeying *N* (*μ*, *σ_i_*^2^).

It can be seen from the formula that, for EEG sequences of the same length, the differential entropy in a frequency band is equivalent to the logarithmic value of its energy in the frequency band.

In order to construct the DE feature matrix, the differential entropy features of five frequency bands (δ [1–4 Hz], θ [4–8 Hz], α [8–12 Hz], β [12–30 Hz], γ [30–50 Hz]) were extracted from EEG samples using a one-second time window. $X^{S}=\left(S_{1}^{S},S_{2}^{S},\ldots ,S_{B}^{S}\right)\in R^{E\times B}$ is defined as the EEG sample containing band B, where E is the number of electrodes 62, *B* (=5) is the number of bands, and $S_{b}^{S}=\left(s_{b}^{1},s_{b}^{2},\ldots ,s_{b}^{E}\right)\in R^{E}\left(b\in \left\{1,2,\ldots ,B\right\}\right)$ represents the EEG of all electrodes collected in band *b*. Then, $S_{b}^{S}$ will be converted into a two-dimensional DE feature matrix $M_{b}^{S}\in R^{H\times W}$, as shown in the following formula:

(3) $$M_{b}^{S}=\left[\begin{array}{ccccccccc} 0 & 0 & 0 & s_{b}^{1} & s_{b}^{2} & s_{b}^{3} & 0 & 0 & 0\\ 0 & 0 & 0 & s_{b}^{4} & 0 & s_{b}^{5} & 0 & 0 & 0\\ s_{b}^{6} & s_{b}^{7} & s_{b}^{8} & s_{b}^{9} & s_{b}^{10} & s_{b}^{11} & s_{b}^{12} & s_{b}^{13} & s_{b}^{14}\\ s_{b}^{15} & s_{b}^{16} & s_{b}^{17} & s_{b}^{18} & s_{b}^{19} & s_{b}^{20} & s_{b}^{21} & s_{b}^{22} & s_{b}^{23}\\ s_{b}^{24} & s_{b}^{25} & s_{b}^{26} & s_{b}^{27} & s_{b}^{28} & s_{b}^{29} & s_{b}^{30} & s_{b}^{31} & s_{b}^{32}\\ s_{b}^{33} & s_{b}^{34} & s_{b}^{35} & s_{b}^{36} & s_{b}^{37} & s_{b}^{38} & s_{b}^{39} & s_{b}^{40} & s_{b}^{41}\\ s_{b}^{42} & s_{b}^{43} & s_{b}^{44} & s_{b}^{45} & s_{b}^{46} & s_{b}^{47} & s_{b}^{48} & s_{b}^{49} & s_{b}^{50}\\ 0 & s_{b}^{51} & s_{b}^{52} & s_{b}^{53} & s_{b}^{54} & s_{b}^{55} & s_{b}^{56} & s_{b}^{57} & 0\\ s_{b}^{58} & 0 & 0 & s_{b}^{59} & s_{b}^{60} & s_{b}^{61} & 0 & 0 & s_{b}^{62} \end{array}\right]$$

where $M_{b}^{S}$ is the 9 × 9 matrix we constructed, other positions in the same matrix are set to 0, and normalization processing is carried out.

**Figure 4:**
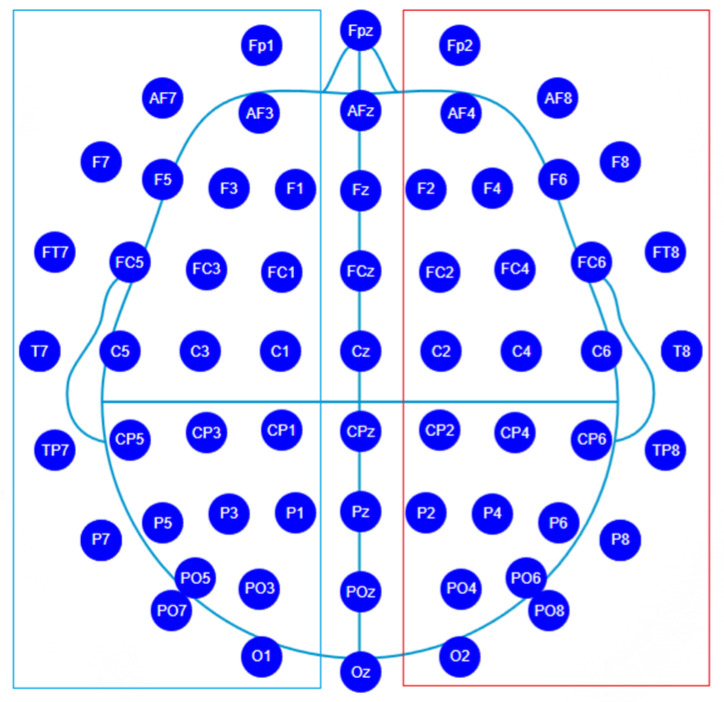
Electrode distribution for symmetrical features.

$M_{t}^{T}$ and $M_{b}^{S}$ represents the 2-D matrix of the original signal and DE after we flattened the electrodes to a 9 × 9 map. Symmetry subtraction refers to the difference between the feature values of the left and right brain symmetrical electrodes. In the 10–20 system, there are 27 pairs of symmetrical electrodes, as shown in Figure 4. Left brain electrodes are in the blue frame, right brain electrodes are in the red frame, and the middle electrode is removed during data processing.

We find the 27 pairs of symmetric electrodes and construct the subtract symmetric matrix using the following formula:

(4) $$d_{t}^{i}=\left\{\begin{array}{c} o_{t}^{i}-o_{t}^{i+27},\;if\;i<27\\ o_{t}^{i}-o_{t}^{i-27},\;if\;i\geq 27 \end{array}\right.\;\mathrm{or}\;d_{b}^{i}=\left\{\begin{array}{c} s_{b}^{i}-s_{b}^{i+27},\;if\;i<27\\ s_{b}^{i}-s_{b}^{i-27},\;if\;i\geq 27 \end{array}\right.$$

where $d_{t}^{i}$ represents the electrode pair difference corresponding to the sampling point $l\in \left[1,f\right]$, $d_{b}^{i}$ represents the electrode pair difference corresponding to the sampling point in frequency band *b* $\in \left[1,B\right]$, and i represents the electrode serial number after removing the channel in the middle position.

The subtract symmetric matrix (SSM) based on the original signal and DE are respectively constructed by the above methods, as shown in the following formula:

(5) $${OS}_{t}=\left[\begin{array}{ccccccccc} 0 & 0 & 0 & d_{t}^{1} & 0 & d_{t}^{28} & 0 & 0 & 0\\ 0 & 0 & 0 & d_{t}^{2} & 0 & d_{t}^{29} & 0 & 0 & 0\\ d_{t}^{3} & d_{t}^{4} & d_{t}^{5} & d_{t}^{6} & 0 & d_{t}^{33} & d_{t}^{32} & d_{t}^{31} & d_{t}^{30}\\ d_{t}^{7} & d_{t}^{8} & d_{t}^{9} & d_{t}^{10} & 0 & d_{t}^{37} & d_{t}^{36} & d_{t}^{35} & d_{t}^{34}\\ d_{t}^{11} & d_{t}^{12} & d_{t}^{13} & d_{t}^{14} & 0 & d_{t}^{41} & d_{t}^{40} & d_{t}^{39} & d_{t}^{38}\\ d_{t}^{15} & d_{t}^{16} & d_{t}^{17} & d_{t}^{18} & 0 & d_{t}^{45} & d_{t}^{44} & d_{t}^{43} & d_{t}^{42}\\ d_{t}^{19} & d_{t}^{20} & d_{t}^{21} & d_{t}^{22} & 0 & d_{t}^{49} & d_{t}^{48} & d_{t}^{47} & d_{t}^{46}\\ 0 & d_{t}^{23} & d_{t}^{24} & d_{t}^{25} & 0 & d_{t}^{52} & d_{t}^{51} & d_{t}^{50} & 0\\ d_{t}^{26} & 0 & 0 & d_{t}^{27} & 0 & d_{t}^{54} & 0 & 0 & d_{t}^{53} \end{array}\right]\;\mathrm{or}{DS}_{b}=\left[\begin{array}{ccccccccc} 0 & 0 & 0 & d_{b}^{1} & 0 & d_{b}^{28} & 0 & 0 & 0\\ 0 & 0 & 0 & d_{b}^{2} & 0 & d_{b}^{29} & 0 & 0 & 0\\ d_{b}^{3} & d_{b}^{4} & d_{b}^{5} & d_{b}^{6} & 0 & d_{b}^{33} & d_{b}^{32} & d_{b}^{31} & d_{b}^{30}\\ d_{b}^{7} & d_{b}^{8} & d_{b}^{9} & d_{b}^{10} & 0 & d_{b}^{37} & d_{b}^{36} & d_{b}^{35} & d_{b}^{34}\\ d_{b}^{11} & d_{b}^{12} & d_{b}^{13} & d_{b}^{14} & 0 & d_{b}^{41} & d_{b}^{40} & d_{b}^{39} & d_{b}^{38}\\ d_{b}^{15} & d_{b}^{16} & d_{b}^{17} & d_{b}^{18} & 0 & d_{b}^{45} & d_{b}^{44} & d_{b}^{43} & d_{b}^{42}\\ d_{b}^{19} & d_{b}^{20} & d_{b}^{21} & d_{b}^{22} & 0 & d_{b}^{49} & d_{b}^{48} & d_{b}^{47} & d_{b}^{46}\\ 0 & d_{b}^{23} & d_{b}^{24} & d_{b}^{25} & 0 & d_{b}^{52} & d_{b}^{51} & d_{b}^{50} & 0\\ d_{b}^{26} & 0 & 0 & d_{b}^{27} & 0 & d_{b}^{54} & 0 & 0 & d_{b}^{53} \end{array}\right]$$

The symmetry quotient feature is the quotient of the feature values of the left and right brain symmetric electrodes. The same as the SSM, it is necessary to find out the symmetric electrodes and eliminate the electrodes in the middle position. Use the following formula to construct the symmetric quotient matrix:

(6) $$q_{t}^{i}=\left\{\begin{array}{c} \frac{o_{t}^{i}}{o_{t}^{i+27}},\;if\;i<27\\ \frac{o_{t}^{i}}{o_{t}^{i-27}},\;if\;i\geq 27 \end{array}\right.\;\mathrm{or}\;q_{b}^{i}=\left\{\begin{array}{c} \frac{s_{b}^{i}}{s_{b}^{i+27}},\;if\;i<27\\ \frac{s_{b}^{i}}{s_{b}^{i-27}},\;if\;i\geq 27 \end{array}\right.$$

where $q_{t}^{i}$ represents the electrode pair quotient value corresponding to the sampling point $l\in \left[1,f\right]$,$q_{b}^{i}$ represents the electrode pair quotient value corresponding to the sampling point in frequency band *b*$\in \left[1,B\right]$*,* and i represents the electrode serial number after removing the channel in the middle position.

According to (6), the quotient symmetric matrix (QSM) based on the original signal and DE show in the following formula:

(7) $${OQ}_{t}=\left[\begin{array}{ccccccccc} 0 & 0 & 0 & q_{t}^{1} & 0 & q_{t}^{28} & 0 & 0 & 0\\ 0 & 0 & 0 & q_{t}^{2} & 0 & q_{t}^{29} & 0 & 0 & 0\\ q_{t}^{3} & q_{t}^{4} & q_{t}^{5} & q_{t}^{6} & 0 & q_{t}^{33} & q_{t}^{32} & q_{t}^{31} & q_{t}^{30}\\ q_{t}^{7} & q_{t}^{8} & q_{t}^{9} & q_{t}^{10} & 0 & q_{t}^{37} & q_{t}^{36} & q_{t}^{35} & q_{t}^{34}\\ q_{t}^{11} & q_{t}^{12} & q_{t}^{13} & q_{t}^{14} & 0 & q_{t}^{41} & q_{t}^{40} & q_{t}^{39} & q_{t}^{38}\\ q_{t}^{15} & q_{t}^{16} & q_{t}^{17} & q_{t}^{18} & 0 & q_{t}^{45} & q_{t}^{44} & q_{t}^{43} & q_{t}^{42}\\ q_{t}^{19} & q_{t}^{20} & q_{t}^{21} & q_{t}^{22} & 0 & q_{t}^{49} & q_{t}^{48} & q_{t}^{47} & q_{t}^{46}\\ 0 & q_{t}^{23} & q_{t}^{24} & q_{t}^{25} & 0 & q_{t}^{52} & q_{t}^{51} & q_{t}^{50} & 0\\ q_{t}^{26} & 0 & 0 & q_{t}^{27} & 0 & q_{t}^{54} & 0 & 0 & q_{t}^{53} \end{array}\right]\;\mathrm{or}\;{DQ}_{b}=\left[\begin{array}{ccccccccc} 0 & 0 & 0 & q_{b}^{1} & 0 & q_{b}^{28} & 0 & 0 & 0\\ 0 & 0 & 0 & q_{b}^{2} & 0 & q_{b}^{29} & 0 & 0 & 0\\ q_{b}^{3} & q_{b}^{4} & q_{b}^{5} & q_{b}^{6} & 0 & q_{b}^{33} & q_{b}^{32} & q_{b}^{31} & q_{b}^{30}\\ q_{b}^{7} & q_{b}^{8} & q_{b}^{9} & q_{b}^{10} & 0 & q_{b}^{37} & q_{b}^{36} & q_{b}^{35} & q_{b}^{34}\\ q_{b}^{11} & q_{b}^{12} & q_{b}^{13} & q_{b}^{14} & 0 & q_{b}^{41} & q_{b}^{40} & q_{b}^{39} & q_{b}^{38}\\ q_{b}^{15} & q_{b}^{16} & q_{b}^{17} & q_{b}^{18} & 0 & q_{b}^{45} & q_{b}^{44} & q_{b}^{43} & q_{b}^{42}\\ q_{b}^{19} & q_{b}^{20} & q_{b}^{21} & q_{b}^{22} & 0 & q_{b}^{49} & q_{b}^{48} & q_{b}^{47} & q_{b}^{46}\\ 0 & q_{b}^{23} & q_{b}^{24} & q_{b}^{25} & 0 & q_{b}^{52} & q_{b}^{51} & q_{b}^{50} & 0\\ q_{b}^{26} & 0 & 0 & q_{b}^{27} & 0 & q_{b}^{54} & 0 & 0 & q_{b}^{53} \end{array}\right]$$

In order to avoid the influence of too large a numerical difference on the subsequent processing, we carried out normalization processing on each two-dimensional matrix constructed.

### 3.3. Classification Network Construction

In the previous section, we introduced the extraction process of the feature matrix, and in this section, we focus on the proposed model. The model proposed by us takes four dimensions as the input of the feature matrix. Firstly, through the feature fusion network, the fusion feature matrix of dimension (200, 64, 64) is obtained. Then, the fusion feature matrix is fed into the multi-axis attention module which is composed of the global attention module and local attention module. Finally, the output of the global attention module and the local attention module are fused again and fed into the classification network to get the classification results of the model. The model proposed in this paper is introduced in the following two parts: feature fusion network and classification model.

#### 3.3.1. Feature Fusion Network

Different features contain different emotional information. In order to consider the complementarity of multiple features, four different feature matrices are sent into the feature fusion network for feature fusion. The fusion network mainly includes 1 × 1 convolution layer, normalization layer, and ReLU activation function. The convolution of 1 × 1 can unify the channel dimensions without changing the dimension of the eigenmatrix, and the activation function can solve the problem of insufficient linear model capability and possible gradient explosion. In addition, in order to capture more details of the feature matrix and optimize the classification effect, we used cubic spline interpolation on the matrix [26]. The matrix dimension based on the original signal is (200, 64, 64), and the matrix dimension based on DE is (5, 64, 64). By up-sample and down-sample, respectively, the dimensions of the four characteristic matrices are unified to (50, 64, 64).

Finally, the four processed feature matrices were spliced to obtain the fused feature matrix (200 × 64 × 64), where 200 corresponds to the number of samples, and 64 × 64 is the size of the feature map after interpolation. The fused feature matrix was used as the input of the classification network.

#### 3.3.2. Classification Network

**Figure 5:**
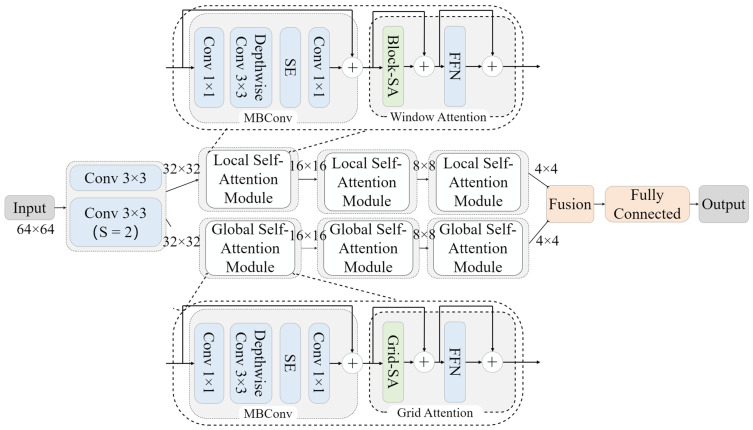
Structure of multi-axis self-attentive model. We propose a model that utilizes four dimensions as the input for the feature matrix. Firstly, the feature fusion network generates the fusion feature matrix with a dimension of (200, 64, 64). Subsequently, the multi-axis attention module receives the fusion feature matrix as input. Following the three-layer attention module, the feature matrix’s dimension transforms to (4, 4). The output feature vector obtained from the fusion of the feature matrix in the local attention module and global attention module is used for classification.

To combine local and global features, global self-attention [27] is taken into account and quadratic complexity is reduced. We introduced a new multi-axis self-attention module. By simply decomposing the spatial axis, full-size attention is decomposed into two sparse forms: local and global. Local and global space can be executed in a single block before interaction, effectively combining local and global features. MBConv was first proposed by Howard [28]. The convolution of linear bottleneck is an inversion layer, which consists of depth-wise separable convolution, a squeeze-excitation layer (SE), and ordinary convolution. Depth-separable convolution can reduce model parameters, thus improving model efficiency [29]. At the same time, it can be regarded as conditional position coding, which causes the model to have a clear position coding layer, and the size is 3×3. General convolution is applied to fully extract the feature information in each input channel to complete the complementary information extraction between multiple channels. Figure 5 shows the network structure. We will go into detail about the composition of each module next.

At first, the network has two layers of convolutional networks to capture the feature information adequately, and then it enters the local self-attention module and the global self-attention module, respectively, in parallel. Finally, the two are integrated and output classification after full connection. Local attention is realized by window attention. For the input eigenmatrix ${X\in R}^{H_{1}\times W_{1}\times C}$, it is converted into a shape tensor $(H_{1}/P\times p$, $W_{1}/P\times p,C$) to represent the windows that are divided into non-overlapping ones, and $(H_{1}/P$×$W_{1}/P$, P×P,C) is obtained, where the size of each window is P × P, and there are ${\left(H_{1}W_{1}/P\right)}^{2}$ total windows. Finally, self-attention calculation is applied to each window, and the original shape is restored after calculation.

**Figure 6:**
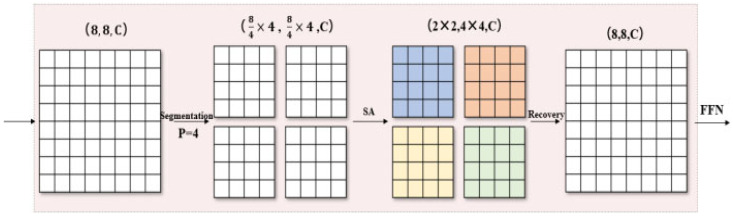
Window attention mechanism.

As shown in Figure 6, we set the $H_{1}$ and $W_{1}$ to 8, and P to 4 to facilitate the process, with the same color representing mixing in space through the self-attention operation.

For global attention, inspired by window attention, we cite a simple and effective way to obtain sparse global attention—grid attention. Instead of using a fixed window size to divide the feature matrix graph, we used a G×G fixed uniform grid of A to transform the tensor grid into shape (G×G, $H_{2}/G\times W_{2}/G$, C), to obtain a window $H_{2}/G\times W_{2}/G$ with an adaptive size. Finally, we use self-attention calculation on G×G, thus indirectly realizing the global interaction. In this way, no matter how the height and width of the input feature matrix graph change, our final feature graph will only divide the specified windows in space, which will reduce the amount of computation. The original shape was restored after calculation.

**Figure 7:**
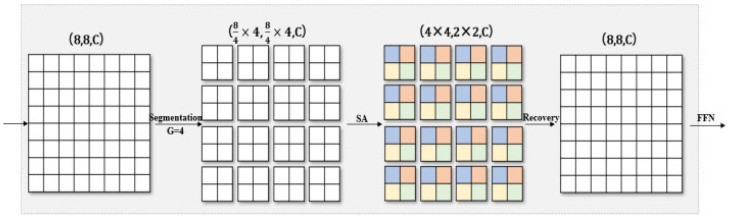
Grid attention mechanism.

The calculation process is shown in Figure 7. As with window attention, H_2_ and W_2_ in the figure are set to 8, and the hyper-parameter G is set to 4. By using the same fixed window and grid size (P = G = 4), you can balance the operations between local and global, both of which have only linear complexity in terms of space size and sequence length. We put inverted mobile bottleneck convolution in front of each attention mechanism, to further improve the generalization and ability of the network model.

## 4. Results

In this section, we conducted subject-dependent experiments to verify the emotion classification performance of the model based on EEG datasets of hearing-impaired subjects and non-hearing-impaired subjects. The training set and test set were divided in a ratio of 7:3. The experimental platform is NVIDIA GeForce RTX 3050 Ti Laptop GPU. The super parameter settings of the classification model are shown in Table 2.

In the three-classification task, happy was classified as a positive emotion, neutral as a neutral emotion, and sad as a negative emotion. Table 3 shows the results of the emotion classification of the subjects of hearing-impaired subjects based on the multi-axis self-attention network model. Subject 9 achieved the highest classification performance in both three-classification (74.78%) and five-classification (53.76%), while subject 1 had the worst classification performance with accuracies of 67.32% and 47.25%. The average accuracy of the proposed method reached 70.72% in the three-classification and 50.15% in the five-classification.

Table 4 lists the EEG signals of 20 non-hearing-impaired subjects during the experiment. Similarly, the emotion classification performance of the network model has also achieved a good performance, with an average accuracy of 72.05% for the three-classification and 51.53% for the five-classification. Subject 11 achieved the highest classification performance in three-classification (75.47%), while subject 10 had the worst classification performance (69.77%). Subject 14 achieved the highest accuracy in five-classification (53.88%), while subject 5 had the worst classification performance (48.79%).

Compared with hearing-impaired subjects, the emotion classification effect of non-hearing-impaired subjects is better. It can be speculated that this result may be related to the difficulty of facial emotion recognition of hearing-impaired subjects under the influence of physiological factors and social environment factors, which is the same as the conclusion of previous researchers [30,31].

To study the classification performance of each emotion based on the multi-axis self-attention network model, we used the confusion matrix. Each row of the confusion matrix represents the real category of data, and each column represents the predicted category.

**Figure 8:**
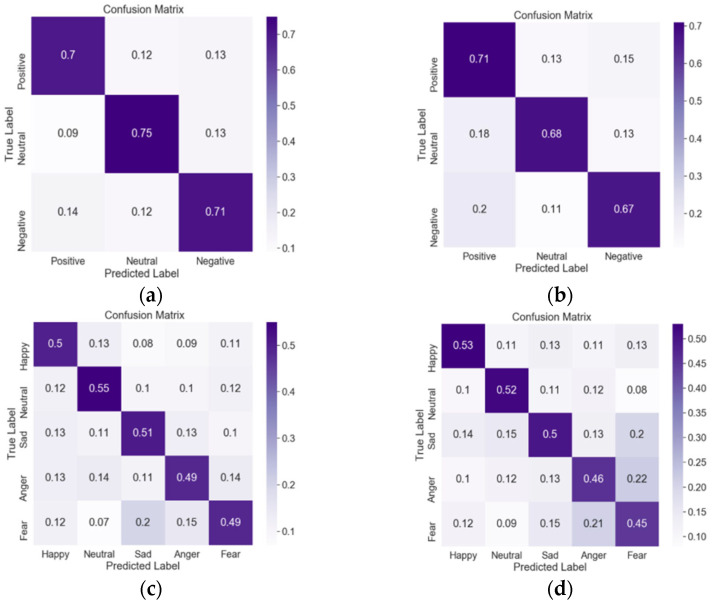
Confusion matrices for non—hearing—impaired subjects and hearing—impaired subjects in two classification tasks. (**a**) Three—classification of non-hearing-impaired subjects. (**b**) Three—classification of hearing-impaired subjects. (**c**) Five—classification of non—hearing-impaired subjects. (**d**) Five-classification of hearing—impaired subjects.

The results of emotion classification are shown in Figure 8. Firstly, we analyzed the confusion matrix of three-classification. By comparing Figure 8a,b, it can be seen that the positive emotion recognition effect of non-hearing-impaired subjects and hearing-impaired subjects is similar. It can be seen from Figure 8a that non-hearing-impaired subjects have the best neutral emotion recognition effect, and Figure 8b shows that hearing-impaired subjects have the best positive emotion recognition effect. Compared with Figure 8a,b, hearing-impaired subjects have slightly better positive emotion recognition effect than non-hearing-impaired subjects, while neutral and negative emotion recognition effect is poor.

In the five-classification tasks, the neutral emotion classification effect of non-hearing-impaired subjects was still the best, and the happy emotion recognition effect of hearing-impaired subjects was the best. By comparison with Figure 8c,d, compared with non-hearing-impaired subjects, hearing-impaired subjects have a better recognition effect on happy emotions, while neutral, sad, angry, and fear recognition effect is lower than non-hearing-impaired subjects. In addition, 22% of the hearing-impaired subjects misclassified anger as fear and 21% misclassified fear as anger. Therefore, it can be speculated that hearing-impaired subjects may have difficulty recognizing anger and fear.

Compared with the hearing-impaired subjects, the non-hearing-impaired subjects are better in the identification ability of negative emotion (sadness, anger, fear), 51%, 49%, and 49%, respectively. The difference in fear emotion classification between hearing-impaired subjects and non-hearing-impaired subjects was the largest, with a difference of 4%. Therefore, it is speculated that, compared with non-hearing-impaired subjects, hearing-impaired subjects have difficulty in recognizing negative facial emotions, which is consistent with our previous video-based study.

## 5. Discussion

To verify the effectiveness of the proposed feature selection method, the comparison experiments of different feature matrix combinations were carried out by both hearing-impaired subjects and non-hearing-impaired subjects. The results appear in Table 5.

The SSM(OEF) and QSM(OEF) represent the subtract symmetric feature matrix and the quotient symmetric matrix based on the original EEG signal. SSM(DE) and QSM(DE) represent the symmetric difference feature and the symmetric quotient feature based on the differential entropy feature. The accuracy of the three-classification and five-classification tasks of the hearing impaired and non-hearing-impaired subjects by using DE features are close to that of SSM(OEF) and QSM(OEF). Compared with SSM(OEF) and QSM(OEF), SSM(DE) and QSM(DE) have better performance in all classification tasks. Concretely, QSM(DE) has the best performance in single feature classification, SSM(DE) + QSM(DE) has the best performance when two feature matrices are fused, and SSM(OEF) + QSM(OEF) + SSM(DE) has the best classification effect when three feature matrices are fused in both three-classification and five-classification tasks. Moreover, the fusion of four feature matrices obtains the best performance. The average classification accuracy of the three-classification tasks and the five-classification tasks reached 70.2% and 50.15%, and 72.05% and 51.53% for hearing-impaired subjects and non-hearing-impaired subjects, respectively. It can be inferred that the features in different domains may be complementary in EEG emotion recognition.

To reflect the effectiveness of the feature fusion matrices we chose a variety of simple machine learning methods (SVM (Linear), GNB (Default), KNN (N = 5), ResNet18) to conduct experiments on the proposed fusion feature matrix SSM(OEF) + QSM(OEF) + SSM(DE) + QSM(OEF), and the results are shown in Table 6. The proposed method achieves outstanding performance. In this work, window attention and network attention are used for local interaction and global interaction, respectively. It may be useful for emotional representation information extraction.

**Figure 9:**
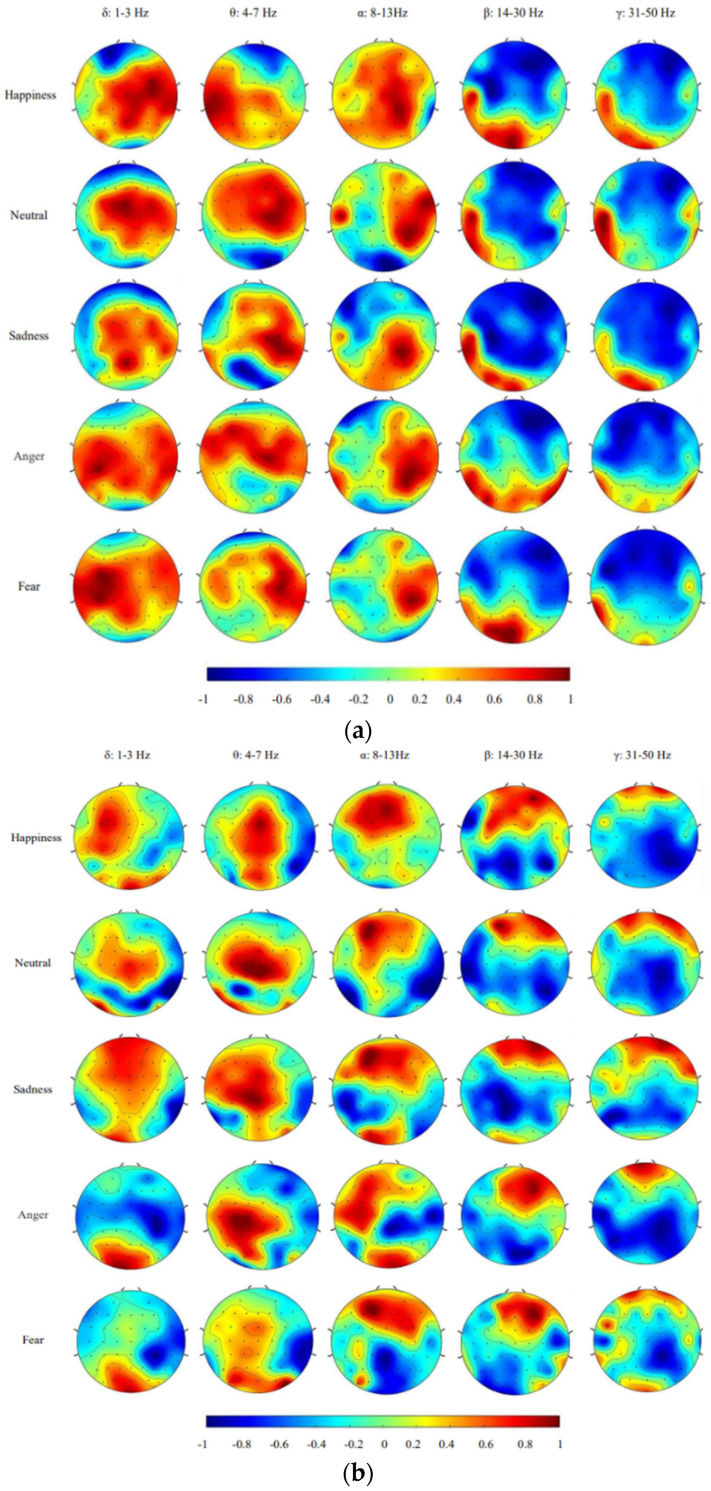
Average brain topographic map based on DE in different frequency bands. (**a**) Hearing—impaired subjects. (**b**) Non-hearing-impaired subjects.

In order to further explore the key regions affecting the emotions of hearing-impaired subjects, we drew brain topographic maps with different frequency bands based on differential entropy features. As Figure 9 shows, by comparing brain topographic maps of different emotions, it was observed that the energy of different emotions was different in the frontal, temporal, parietal, and occipital lobes. The negative emotion was identified by the changes near the temporal lobe, while the happy emotion was identified by the parietal lobe and the right temporal lobe. In contrast, positive emotion was identified by the occipital lobe and temporal lobe.

In the process of recognizing anger and fear, it can be seen that the key areas identified by hearing-impaired subjects overlap, which is different from that of non-hearing-impaired subjects. For anger and fear, there was no significant overlap between the activated electrodes of the hearing impaired and non-hearing-impaired subjects across the five bands. The hearing-impaired subjects activated a large number of brain regions in the δ and θ bands, while the non-hearing-impaired subjects only had a small number of activated electrodes in the occipital and parietal lobes. In the latter three frequency bands, the overlap rate of the activated electrode regions between the hearing-impaired and non-hearing-impaired subjects was also very low, which partly explains why the hearing-impaired subjects were less effective in recognizing anger and fear than the non-hearing-impaired subjects. This is related to the bias of hearing-impaired subjects in recognizing negative emotions. The distribution of emotion-discriminating brain area in the temporal lobe of hearing-impaired subjects was the same as that of non-hearing-impaired subjects.

The difference, however, is that the discriminative brain regions of the hearing-impaired subjects are also located in the parietal lobe. According to studies of brain function, the parietal lobe is involved in the integration of vision and visual processing. This may indicate that visual information plays a more important role in the emotional discrimination of hearing-impaired subjects due to the loss of hearing ability. In the absence of auditory channels, hearing-impaired subjects may use the coordination of multiple brain regions to complete the acquisition and expression of emotional information.

## 6. Conclusions

In this paper, the emotional-faces-induced EEG emotion recognition scheme was proposed. We collected EEG signals from hearing-impaired subjects and non-hearing-impaired subjects when they were watching the different face pictures. To obtain the spatial domain feature, the SSM and QSM were based on the original signals and DE features, respectively, which were reflected as four feature matrices. The classification network model based on the multi-axis self-attention mechanism is used for emotion recognition. The quadratic complexity of ordinary attention is reduced to linearity by using a multi-axis self-attention module without loss of non-locality. The spatial interaction between local and global can be realized in each block, effectively combining local and global characteristics. The promising results were obtained both in hearing-impaired and non-hearing-impaired subjects for three-classification and five-classification tasks with the proposed four-feature matrix fusion strategy. It proves that the complementarity between features can be used to improve the effect of emotion classification.

Based on the classification results of the three and five categories of emotion, the differences in the ability of non-hearing-impaired subjects and hearing-impaired subjects to recognize different emotions and the differences in the electrodes of active brain regions were analyzed in detail through the confusion matrix and brain topographic map. We find that the key areas identified by hearing-impaired subjects overlap and are obviously different from that of non-hearing-impaired subjects in the process of recognizing anger and fear. This is related to the bias of hearing-impaired subjects in recognizing negative emotions. Additionally, we also found that hearing-impaired subjects may use the coordination of multiple brain regions to complete the acquisition and expression of emotional information in the absence of auditory which meets the conclusion of hearing-impaired people in video stimulation.

In future work, we will extend our dataset and focus on developing general feature extraction methods and classification models for hearing-impaired and non-hearing-impaired people.

## Figures and Tables

**Table 1:** Statistics of 20 hearing-impaired subjects.

No.	Gender	Age	Damaged Age	Damage Level	Hearing Aid Equipment
1	Male	21	3	1	Right side
2	Male	21	0	2	Both sides
3	Female	19	innate	1	Both sides
4	Female	21	2	2	Both sides
5	Male	21	2	1	Both sides
6	Male	25	2	3	Both sides
7	Male	21	3	1	Left side
8	Male	21	innate	1	Both sides
9	Male	25	2	1	Right sides
10	Female	20	innate	1	Both sides
11	Male	21	1	1	Both sides
12	Female	25	1	1	Both sides
13	Male	21	3	1	Both sides
14	Male	21	3	1	Both sides
15	Female	21	3	2	Right side
16	Female	21	1	1	Left side
17	Male	22	innate	1	Both sides
18	Male	24	2	1	Both sides
19	Female	20	1	1	Both sides
20	Male	21	3	1	Both sides

**Table 2:** Network parameter Settings.

Hyper-Parameter	Value
Optimizer	SGD
Weights Decay	$1\times 10^{-4}$
Learning Rate	0.01
Learning Rate Decay	0.01
Batch Size	32
Input Size	(9, 9)
Training Epochs	30

**Table 3:** The classification results of hearing-impaired subjects.

Subjects	Accuracy (%)		Recall (%)		F1-Scores (%)	
	Three Classes	Five Classes	Three Classes	Five Classes	Three Classes	Five Classes
1	67.32	47.25	67.31	47.23	69.34	47.29
2	72.51	49.37	72.42	49.38	72.47	49.24
3	72.35	49.77	72.05	49.73	72.33	49.79
4	68.25	48.59	68.27	48.56	68.24	48.51
5	73.21	51.23	73.19	51.24	73.22	51.23
6	71.8	50.48	71.81	50.49	71.79	50.45
7	68.5	48.91	68.48	48.93	68.51	48.88
8	69.9	48.35	69.92	48.31	69.87	48.39
9	74.78	53.76	74.74	53.74	74.79	53.81
10	72.52	52.15	72.5	52.11	72.54	52.2
11	67.39	49.23	67.37	49.28	67.41	49.17
12	68.98	48.36	68.97	48.34	68.98	48.47
13	70.35	50.14	70.33	50.18	70.37	50.11
14	71.25	50.48	71.27	50.43	71.21	50.39
15	69.54	49.37	69.57	49.39	69.54	49.27
16	72.68	52.37	72.66	52.33	72.69	52.43
17	69.58	50.45	69.57	50.43	69.58	50.28
18	71.57	51.34	71.54	51.33	71.59	51.41
19	70.58	51.11	70.59	51.13	70.56	50.86
20	71.39	50.19	71.4	50.19	71.35	50.28
Average	70.72	50.15	70.7	50.14	70.82	50.12

**Table 4:** Emotion classification results of non-hearing-impaired subjects.

Subjects	Accuracy (%)		Recall (%)		F1-Scores (%)	
	Three Classes	Five Classes	Three Classes	Five Classes	Three Classes	Five Classes
1	71.28	49.87	71.32	49.91	71.12	49.67
2	72.44	50.65	72.34	50.41	73.68	50.98
3	74.25	51.23	74.34	51.33	74.17	51.21
4	70.67	50.39	70.52	50.18	71.04	50.51
5	69.89	48.79	70.04	48.99	69.88	48.57
6	72.87	52.48	72.78	51.97	72.86	52.74
7	73.41	51.69	73.24	51.61	73.37	51.71
8	71.43	50.72	71.54	50.79	71.34	50.49
9	73.11	53.71	72.98	53.54	73.26	53.94
10	69.77	50.31	69.87	50.47	69.77	50.08
11	75.47	53.87	75.25	53.71	75.41	53.94
12	72.11	52.48	72.34	52.52	72.05	51.98
13	69.88	51.1	69.94	51.21	69.91	51.11
14	73.64	53.88	73.51	53.79	73.74	53.96
15	71.28	50.77	71.34	50.85	71.15	50.49
16	70.79	50.64	70.62	50.49	70.54	50.83
17	73.84	52.68	73.88	52.71	73.83	52.29
18	71.32	51.43	71.28	50.97	71.51	51.87
19	73.15	52.58	73.24	52.69	73.1	52.35
20	70.38	51.33	70.34	51.3	70.43	51.46
Average	72.05	51.53	72.04	51.47	72.11	51.51

**Table 5:** Comparison of results of different feature combinations.

Feature Combinations	Average Accuracy (%)			
	*HP Three Classes	*HP Five Classes	Non-Hearing-Impaired Three Classes	Non-Hearing-Impaired Five Classes
DE	60.35	42.26	61.04	45.91
SSM(OEF)	60.13	41.87	61.36	45.05
QSM(OEF)	59.97	42.93	61.77	44.97
SSM(DE)	62.71	44.79	62.35	46.33
QSM(DE)	63.74	43	63.98	46.7
SSM(OEF) + QSM(OEF)	65.37	47.03	65.56	48.33
SSM(DE) + QSM(DE)	67.83	47.84	68.37	48.72
SSM(OEF) + SSM(DE)	64.75	45.03	65.06	46.89
SSM(OEF) + QSM(DE)	66.3	45.87	67.17	47.14
QSM(OEF) + SSM(DE)	65.17	45.86	66.04	47.02
QSM(OEF) + QSM(DE)	64.71	45.13	66.37	46.97
SSM(OEF) + QSM(OEF) + SSM(DE)	68.9	48.65	71.2	50.89
SSM(OEF) + QSM(OEF) + QSM(DE)	68.77	48.2	70.83	49.97
SSM(OEF) + SSM(DE) + QSM(DE)	68.37	47.97	70.16	50.33
QSM(OEF)+ SSM(DE) + QSM(DE)	68.71	48.53	70.88	50.72
SSM(OEF) + QSM(OEF) + SSM(DE) + QSM(DE)	70.2	50.15	72.05	51.53

*HP stands for hearing-impaired subjects.

**Table 6:** The comparison results of the proposed method and other algorithms.

Model	Average Accuracy (%)			
	HP Three Classes	HP Five Classes	Non-Hearing-Impaired Three Classes	Non-Hearing-Impaired Five Classes
SVM (Linear)	64.24	45.78	66.47	47.45
GNB (Default)	63.06	42.87	64.36	45.05
KNN (N = 5)	63.35	43.26	65.04	45.91
ResNet18	67.85	48.37	69.26	49.75
Our	70.2	50.15	72.05	51.53

## Data Availability

The data is unavailable due to privacy or ethical restrictions.

## Supplementary Materials

The following supporting information can be downloaded at: https://www.mdpi.com/article/10.3390/s23125461/s1.
