# Supplementary material for: Image-Evoked Emotion Recognition for Hearing-Impaired Subjects with EEG Signals
Source: Sensors (Basel). 2023 Jun 9;23(12):5461. doi: 10.3390/s23125461 (PMC10301379; doi:10.3390/s23125461)
Supplement: Supplementary file 1 [file sensors-23-05461-s001.zip › sensors-2427249-supplementary.pdf]

Basic information questionnaire of experimental subjects

Dear students,

Hello!

We are researchers from the School of Electrical Engineering and Automation, Tianjin University of Technology. In order to understand the emotional state of hearing-impaired adults and to study how to adopt effective measures to help them improve their expressive ability, we hereby investigate your following information. Please be assured that we will not divulge any of your personally identifiable information and all data collected is for research purposes only. Thank you for your cooperation!

Name:	Student number:	Gender:
Age:	Date of birth:	Native place:
Grade:	Major:	Source of student:
Phone number:		
What is the cause of your hearing loss?		
Congenital Specific reason:		
Acquired Specific reason:		
Acquired hearing loss time:	Age at that time:	
Hearing damage level:		
Degree of hearing loss(db) left ear: right ear:		
Whether to wear an artificial hearing aid:		
If yes, your wearing position is : left ear: right ear: all:		
How long you wear your hearing aid:	Age at that time:	
Frequency of hearing aid use:		
Have you studied in ordinary schools before entering university?<br>Which state?	Have you learned to lip-read?	
Do you know sign language?	Can you speak?	
Have you had any formal speech rehabilitation training?<br>When did it take place?<br>How long did it last?		
Daily communication mode:<br>Sign language Speaking Both	The proportion of spoken:<br>The proportion of sign language:	
